# Sleep quality profiles related to cognitive impairment

**DOI:** 10.1002/alz70861_108358

**Published:** 2025-12-23

**Authors:** Alana Brown, Aline Moussard, Andrew Lim, Jason Steffener, Sylvie Belleville, Malcolm Binns, Nicole D. Anderson, the Consortium for the Early Identification of Alzheimer's Disease‐Quebec (CIMA‐Q) and the Comprehensive Assessment on Neurodegeneration and Dementia (COMPASS‐ND) group

**Affiliations:** ^1^ Rotman Research Institute, Baycrest Academy for Research and Education, Toronto, ON Canada; ^2^ Institut Universitaire de Gériatrie de Montréal, Montréal, QC Canada; ^3^ Université de Montréal, Montréal, QC Canada; ^4^ Sunnybrook Health Sciences Centre, Toronto, ON Canada; ^5^ University of Toronto, Toronto, ON Canada; ^6^ University of Ottawa, Ottawa, ON Canada; ^7^ Dalla Lana School of Public Health, University of Toronto, Toronto, ON Canada

## Abstract

**Background:**

Females have a higher risk of developing Alzheimer’s disease (AD) and report poorer sleep quality with age compared to males. Because poor sleep quality is also a risk factor for AD, it may contribute to sex‐specific differences in AD risk. Females differ in the prevalence, presentation, and severity of sleep disorders, and tend to show greater mismatch between objective and subjective sleep metrics. To better understand these factors, this study investigated sex differences in self‐reported sleep quality metrics across varying levels of cognitive impairment.

**Method:**

Cross‐sectional self‐report data from COMPASS‐ND/CIMA‐Q were analyzed across three groups [cognitively unimpaired (CU, n=104), subjective cognitive impairment (SCI, n=108), amnestic mild cognitive impairment (MCI, n=290)], including components of the Pittsburgh Sleep Quality Index, metrics of daytime fatigue/dysfunction, sleep medication use, sleep apnea, rapid eye movement (REM) sleep behaviour disorder, and restless leg syndrome. Principal component analyses including these sleep variables were run separately for males (*n* =246) and females (*n* =256). Linear models assessed whether principal component scores differed by sex and group (CU, SCI, MCI).

**Result:**

In both sexes, the first principal component (PC1) reflected overall sleep quality and daytime fatigue/dysfunction, with higher scores indicating better sleep and lower fatigue/dysfunction. The second component (PC2) captured discordance between sleep quality and fatigue, with higher scores indicating poor sleep but low fatigue. Linear models showed that both PC1 and PC2 scores were significantly lower in MCI compared to CU. Thus, MCI was associated with both poor sleep and high fatigue (low PC1), as well as good sleep but high fatigue (low PC2), suggesting a mismatch between perceived sleep quality and daytime functioning for some participants. While no significant sex‐by‐group interactions were found, subtle sex differences were noted: Compared to males, females with low PC2 scores reported more symptoms of REM sleep behaviour disorder and restless leg syndrome.

**Conclusion:**

Findings highlight distinct sleep and cognitive impairment patterns, with potential sex‐specific sleep symptom profiles. Understanding these profiles could improve early identification of cognitive decline and support targeted sleep interventions. Tailoring approaches to these differences may enhance both sleep quality and cognitive outcomes in aging populations.

